# Ultrasound-Indicated Cerclage in Twin Pregnancies: A Cohort Study

**DOI:** 10.1155/2022/9450141

**Published:** 2022-11-30

**Authors:** Suyeon Park, Young-Eun Lee, Keun-Young Lee, Ji-Eun Song

**Affiliations:** ^1^Department of Obstetrics and Gynecology, University of Hallym College of Medicine, Hallym Sacred Heart Hospital, Anyang, Republic of Korea; ^2^Department of Obstetrics and Gynecology, University of Hallym College of Medicine, Kangnam Sacred Heart Hospital, Seoul, Republic of Korea

## Abstract

**Background:**

To report the pregnancy and neonatal outcomes in patients with twin pregnancies who underwent ultrasound-indicated cerclage (UIC) and to compare them to patients with singleton pregnancies undergoing the same procedures.

**Methods:**

Patients who underwent UIC between January 2010 and December 2020 at Kangnam Sacred Heart Hospital were reviewed. We compared characteristics, pregnancy, and neonatal outcomes between patients with singleton and twin pregnancies.

**Results:**

A total of 94 women (56 singleton and 38 twin pregnancies) underwent UIC were included. The mean gestational age (GA) at cerclage and preoperative cervical length (CL) were not significantly different. Twin pregnancies were more likely to deliver at earlier median gestations than singletons (singleton, 36 + 1 weeks vs twin, 32 + 6 weeks, and *p* = 0.004). The frequency of preterm delivery <34 weeks in twin group was higher than in singleton group (15 (26.8%) vs 20 (52.6%) and *p* =0.016). However, the frequency of preterm delivery <32, <28, and <24 weeks was not significantly different between two groups. Although neonatal weights in singleton pregnancies were heavier than twin pregnancies, neonatal mortality and morbidities were not significantly different between two groups. Among various factors contributing to preterm birth, preoperative CL ≤ 15 mm was independently associated with a higher risk of preterm delivery before 34 weeks. Furthermore, pregnancy and neonatal outcomes of twin pregnancies with cervical length ≤ 15 mm are comparable with those of singleton pregnancies (GA at delivery, singleton, 35 + 1 weeks vs twin, 32 + 5 weeks, and *p* = 0.24; neonatal mortality, singleton, 3.4% vs twin, 4.8%, and *p* = 0.64).

**Conclusion:**

The pregnancy and neonatal outcomes of UIC in twin pregnancies were comparable to those in singleton pregnancies, especially when CL is ≤15 mm. UIC might be considered a safe procedure for twin pregnancies.

## 1. Introduction

The twin birth rate has increased along with the continuous rise in use of assisted reproductive technology, which has been associated with an increased risk of preterm birth, low birth weight, and neonatal complications [1, 2]. Further, the risk of prematurity becomes 7.5 times higher for twin gestations than for singletons [1–3]. In 2018, the incidence of twin births was 32.6 twins per 1,000 births, with 60.32% of twins delivered before 37 weeks of gestation and 19.52% before 34 weeks, among singleton pregnancies, only 8.24% delivered before 37 weeks of gestations and 2.12% before 34 weeks [2]. Owing to the higher rate of preterm birth in twin pregnancies, the risks for low birth weight, neonatal morbidity, and mortality are much higher than those in singleton pregnancies [1, 2].

Cervical insufficiency, defined as painless cervical dilatation in the midtrimester, is a well-known cause of preterm birth, accounting for 10-25% of all pregnancy losses in the second trimester [3, 4]. Singleton pregnancies with short cervical length (CL) (less than 25 mm) occurring before 24 weeks of gestation and prior spontaneous preterm birth at less than 34 weeks of gestation have been associated with poor prognosis [4]. However, use of the treatment ultrasound-indicated cerclage (UIC) is associated with a significant decrease in preterm birth outcomes and improvement in neonatal morbidity and mortality [4].

In twin pregnancies, a short cervix (CL ≤ 25 mm) before 24 weeks of gestation is significantly associated with preterm birth [5, 6], with the risk inversely proportional to cervical length [6–8]. However, data on the efficacy of UIC in twins is limited, and the existing studies have shown various results regarding its efficacy [6, 8–10]. Thus, the aim of our study was to report pregnancy and neonatal outcomes for patients with twin pregnancies who underwent UIC and to compare them to patients with singleton pregnancies having undergone the same procedure.

## 2. Methods

This was a retrospective cohort study of singleton and twin pregnancies in women who had undergone UIC at Kangnam Sacred Heart Hospital, Seoul, Korea, between January 2010 and December 2020. The study protocol followed the guidelines of the Declaration of Helsinki and was approved by the Institutional Review Board of the Kangnam Sacred Heart Hospital (approval no. 2020-01-006). Formal consent was not required due to retrospective nature of the study.

We included singleton and twin pregnancies who presented with painless short cervix (CL ≤ 25 mm) by transvaginal ultrasound sonography between 16 0/7 and 23 6/7 weeks of gestation and who underwent UIC and subsequently delivered at the same hospital. Only singleton patients who had prior spontaneous preterm birth at less than 34 weeks of gestation, which were adequate candidates for UIC were included [4]. We excluded patients who carried monochorionic-monoamniotic twins, had chorioamnionitis, had preterm premature rupture of membranes or persistent contractions, had fetal abnormalities, or had medically indicated preterm births (preeclampsia with severe features, placenta abruption, placenta previa, or twin-twin transfusion syndrome). Clinical chorioamnionitis was diagnosed by one or more of the following criteria: maternal fever ≥ 38°C, maternal or fetal tachycardia, or maternal blood cell count ≥ 15, 000/mm^3^.

Cerclage was performed by one of the two physicians in a single center. In our practice, the techniques for cerclage placement are as follows: McDonald cerclages are performed with a purse-string suture using 5 mm polyester tape (Cervix set, B Braun, Melsungen, Germany). All patients were intravenously administered cephalosporin for 3 days after surgery.

Pregnancy data collected for this study included maternal age, parity, rate of assisted reproductive technology (ART), prior cervical operation, prior preterm birth history, body mass index (BMI), gestational age (GA) at cerclage and delivery, pre and postoperative CL, interval from cerclage to delivery, and rate of preterm birth. Neonatal outcomes collected included birth weight, Apgar score at one and five minutes, rate of neonatal mortality, and neonatal morbidities (respiratory distress syndrome (RDS), bronchopulmonary dysplasia (BPD), necrotizing enterocolitis (NEC), intraventricular hemorrhage (IVH) grade 3 or 4, retinopathy of prematurity (ROP) requiring three more ophthalmology office visits, and sepsis).

We compared pregnancy and neonatal outcomes between the two groups and evaluated the risk factors contributing to preterm birth before 34 weeks of gestation. In addition, we conducted a subgroup analysis according to preoperative CL cut-off values to investigate the efficacy of UIC for certain CL.

Descriptive statistics were calculated with continuous data presented as medians (ranges) and categorical variables as numbers (percentages). Parametric testing was used to compare data with normal distributions, and comparisons were performed using the Fisher's exact or Mann-Whitney *U* test. Multivariable logistic regression was performed to determine the variables associated with the outcomes. A receiver-operating characteristic (ROC) curve was used to obtain the cut-off value for CL, and the area under the curve (AUC) was used as an indicator of accurate prediction. The cut-off value for each parameter was determined according to sensitivity and specificity. Kaplan-Meier survival curves were used to compare gestational age at delivery according to the plurality of gestations. Data were assessed using IBM SPSS Statistics software (version 26.0; IBM Corp., Armonk, NY, USA). *P* values <0.05 were considered to be statistically significant.

## 3. Results

A total of 94 patients met the inclusion criteria. Among them, all singleton (*N* = 56) and twin (*N* = 38) pregnancies were subjected to UIC. Among the 38 twin pregnancies, 33 (86.8%) were dichorionic-diamniotic twins, and five (13.2%) were monochorionic-diamniotic twins. All cases of UIC were surgically successful, and there were no cases of intraoperative membrane rupture or immediate pregnancy loss. The median maternal age was 34 years for singleton pregnancies and 33 years for twin pregnancies. The incidence of nulliparity and in vitro fertilization was higher in twin pregnancies than in singleton pregnancies, and the incidences of prior preterm birth and second trimester loss were lower in twin pregnancies than in singleton pregnancies. GA at cerclage, BMI, incidence of prior cervical operation, use of tocolytics, and vaginal progesterone were not significantly different between the two groups (Table 1).

Table 2 shows pregnancy outcomes between the two groups. Twin pregnancies were more likely to deliver at earlier median gestations than singletons (36 + 1 vs 32 + 6 weeks and *p* = 0.004), and the rate of preterm delivery before 34 weeks of gestation in twin group was higher than that of the singleton group (15 (26.8%) vs 20 (52.6%) and *p* = 0.016). However, the rates of preterm birth before 32, 28, and 24 weeks of gestation were not different between the two groups (Table 2).

The neonatal outcomes of the two groups are shown in Table 3. The median birth weight in singletons was heavier than that of twins, but the incidences of very low birth weight infants (<1,500 g, VLBWI) (25% vs 25% and *p* = 1) and extremely low birth weight infants (<1,000 g, EVLBWI) (5.4% vs 10.5% and *p* = 0.353) were not different between the two groups. In addition, the Apgar score at 5 min under 7, neonatal mortality, and morbidities including RDS, BPD, NEC, IVH, ROP, and sepsis did not differ between the two groups.

**Figure 1:**
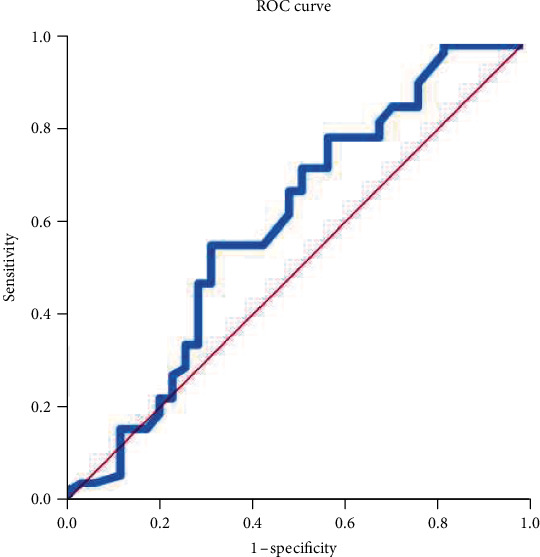
Receiver-operating characteristic curves for cervical length and preterm birth.

To evaluate the risk factors for preterm birth before 34 weeks, we performed ROC curve and logistic regression analyses. The ROC curve for preoperative CL is shown in Figure 1. The AUC value was greater than 0.600 (0.604), and the optimal cut-off value of CL measured at cerclage was 15 mm (sensitivity, 55.9%; specificity, 68.6%). In bivariable analysis, presence of twin pregnancies (odds ratio [OR], 3.037; 95% confidence interval [CI], 1.274–7.242), history of prior preterm birth or second trimester loss (OR, 2.934; 95% CI, 1.227–7.014), and preoperative CL ≤ 15 mm (OR, 2.769; CI, 1.149–6.673) were associated with preterm birth before 34 weeks. Maternal age, parity, ART, prior cervical surgery, BMI, GA at cerclage, and postoperative cervical length were not associated with preterm birth.  Table 4 shows the results of the multivariable regression analysis of the factors associated with preterm birth before 34 weeks. Only a preoperative CL ≤ 15 mm on transvaginal ultrasound was associated with a significantly increased risk of preterm birth before 34 weeks of gestation (OR, 2.870; CI, 1.150-7.164).

Further, we performed a subanalysis for patients with CL ≤ 15 mm, which is the optimal cut-off value associated with preterm birth before 34 weeks. There were no significant differences in the pregnancy and neonatal outcomes between singleton and twin pregnancies with this CL, except for neonatal birth weight (Tables 5 and 6).

**Figure 2:**
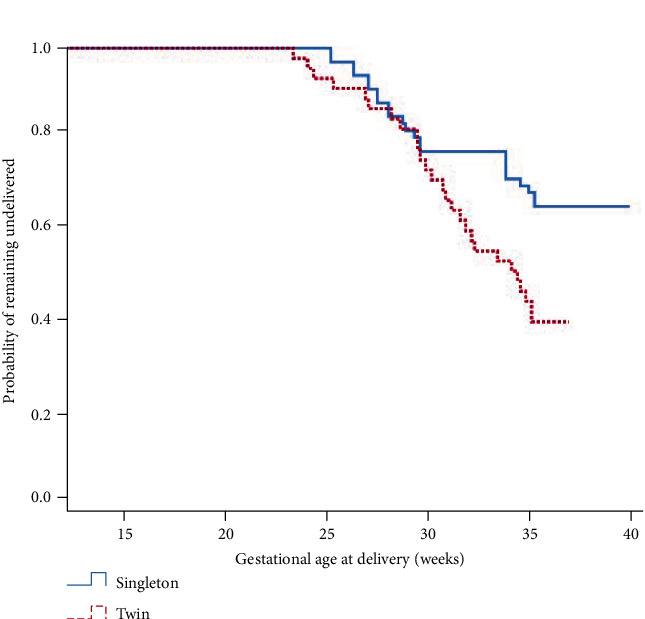
The Kaplan-Meier survival curves on gestational age at delivery between singleton and twin pregnancies (median age at delivery, 36 + 1 weeks vs 32 + 6 weeks, and *p* = 0.006).

**Figure 3:**
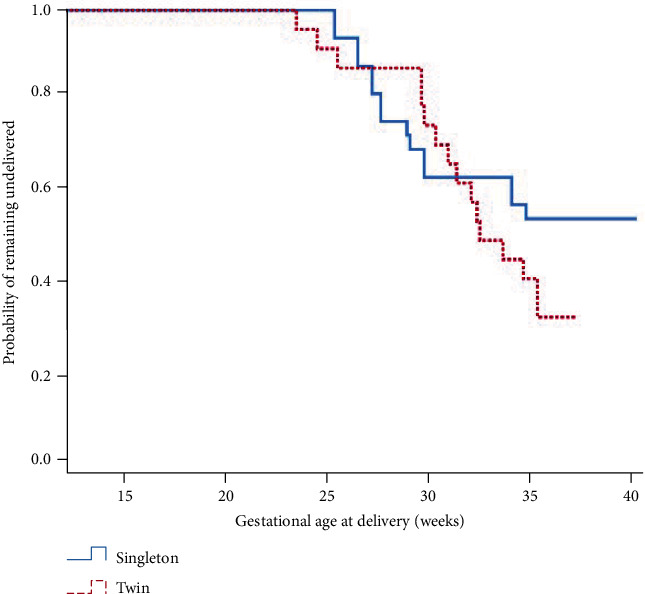
The Kaplan-Meier survival curves on gestational age at delivery between singleton and twin pregnancies with preoperational cervical length ≤ 15 mm (median age at delivery, 35 + 1 weeks vs 32 + 5 weeks, and *p* = 0.218).

Figure 2 shows the Kaplan-Meier curve generated for GA at delivery comparing survival curves between UIC in singleton and twin pregnancies. The median gestational ages at delivery in singleton and twin pregnancies were 36 + 1 and 32 + 6 weeks of gestations, respectively (*p* = 0.006). However, the subgroup of women with preoperative CL ≤ 15 mm had no difference in the Kaplan-Meier curve between the two groups (35 + 1 vs 32 + 5 weeks of gestations, *p* = 0.218) (Figure 3).

## 4. Discussion

Our study demonstrated that UIC in twin pregnancies did not increase the rate of preterm birth before 32, 28, or 24 weeks of gestation, and there were no statistical differences in neonatal outcomes including rates of VLBWI and EVLBWI, Apgar scores at 5 min, neonatal mortality, or morbidities between the two groups. Although the frequency of preterm delivery <34 weeks in twin pregnancies was higher than that in singleton pregnancies (15 (26.8%) vs 20 (52.6%) and *p* = 0.016), it was not the factor of twin pregnancy itself but instead preoperative CL ≤ 15 mm that was associated with preterm delivery before 34 weeks. In addition, benefits of UIC in twin pregnancies were observed in subgroups with preoperative CL ≤ 15 mm similar to those in singleton pregnancies.

Preterm delivery occurs more frequently in twin pregnancies than in singleton pregnancies [1, 2, 11]. In 2015, the US National Vital Statistics System revealed that more than five of every 10 twins were delivered preterm [12]. Our study also showed that twin pregnancies were more likely to deliver at earlier median gestations than singletons (36 + 1 weeks vs 32 + 6 weeks and *p* = 0.004), and the frequency of preterm delivery before 34 weeks of gestation in the twin UIC group was significantly higher than that in the singleton UIC group, with the exception of gestation before 32 weeks. Therefore, we evaluated the risk factors for preterm birth between 32 and 34 weeks. A total of four women delivered between 32 and 34 weeks, all with twin pregnancies and three with preoperative CL ≤ 15 mm. This indicates that it is not only a preoperative CL ≤ 15 mm that may be associated with preterm birth between 32 and 34 weeks but also the twin itself. One plausible explanation for this is that multiple pregnancies have more of an effect on uterine distention and excessive uterine stretch, which are well-known causes of preterm birth than singleton pregnancies.

According to US National Vital Statistics in 2013, the rate of preterm delivery before 32 weeks was 11.3% for twins and 1.5% for singletons. (odd ratio 8.2 and 95% CI: 8.0-8.3) [11]. However, our study found that the frequency of preterm delivery at <32 weeks did not differ between singletons and twins. This emphasizes the findings that UIC in twin pregnancies decreased the rate of preterm birth at <32 weeks and had promising effects similar to those in singleton pregnancies.

In our study, the median neonatal birth weight in twin and singleton pregnancies were 1920 g and 2750 g, respectively (*p* < 0.001). The rate of Apgar score of under 7 at 1 min in twin pregnancies was higher than that in singleton pregnancies. The differences in median neonatal birth weight and Apgar score of under 7 at 1 min may be due to differences in median gestational age at delivery. Because the rate of term delivery in singleton pregnancies was higher than that in twin pregnancies, this may be the cause of these differences. However, the frequencies of VLBWI and EVLBWI, Apgar scores under 7 at 5 min, neonatal mortality, and morbidities did not differ between the two groups. These notable findings may suggest that UIC in twin pregnancies is associated with viable neonatal outcomes and contribute to a higher overall perinatal survival, similar to the findings of some studies [8, 13] and is not as harmful as previously suggested [6, 14].

In addition, we compared neonatal weight using percentile by each nomogram for singleton and twin pregnancies. Because there are no Korean population-based singleton and twin growth chart, we used Fenton 2013 chart for singleton and UK population-based chart for twin pregnancies [15, 16]. The result of comparison using percentile was similar as the result using gram (singleton, 56.8% vs twin, 42.9%, and *p* = 0.003). This may be due to heterogeneity of nationality and lack of each population-based growth chart. In our study, most of patients (85/94) were Korean, but 9 patients were not Korean (5 Chinese, 2 Vietnamese, 1 Thai, and 1 Japanese).

There has been no consensus regarding the efficacy of twin cerclages. A few studies have reported successful outcomes of both ultrasound-indicated and physical examination-indicated cerclages in twin pregnancies like our study [8, 13, 17, 18]. For example, Zanardini et al. evaluated 28 cases of UIC in twin pregnancies at a single center and reported high overall perinatal survival [13]. In addition, a recent systematic review concluded that cerclage is beneficial in reducing preterm birth and prolonging pregnancy in twin pregnancies with CL < 15 mm [17]. However, most previous studies have reported negative or insufficient results regarding the efficacy of UIC in twin pregnancies. According to a meta-analysis using individual patient-level data from three randomized controlled trials by Berghella et al., cerclage for twin pregnancies in mothers with a short cervix showed an increased risk of preterm birth and perinatal mortality compared with the control group who did not receive cerclage [14]. In addition, another meta-analysis by Saccone et al. showed that UIC in twins with CL ≤ 25 mm was not associated with the prevention of preterm birth compared with a control group who did not undergo UIC [19]. Further, a Cochrane review concluded that there was no evidence that cerclage is effective in preventing preterm birth in twin pregnancies owing to the lack of literature data [20]. The reasons for this disagreement on the efficacy of UIC in twin pregnancies may be due to differing surgical techniques and protocols among the various institutions.

In singleton pregnancies, a short CL ≤ 25 mm is thought to be a useful predictor of risk for preterm delivery and indication of UIC [4]. However, this definition is not applicable to twin pregnancies because of the different mechanisms that cause cervical shortening. According to a previous study by Souka et al., the rate of preterm birth under 32 weeks of gestation increases exponentially with decreased cervical length at 23 weeks of gestation in twin pregnancies [21]. Thus, CL plays a significant role in predicting preterm births in twin pregnancies. For these reasons, we used ROC curves to determine the optimal cut-off value of CL to predict preterm delivery at <34weeks.

Our study suggested an optimal cut-off value of preoperative CL (15 mm) for UIC in twin pregnancies through ROC curve analysis and showed that UIC with preoperative CL ≤ 15 mm in twin pregnancies had comparable effectiveness in pregnancy outcomes, neonatal outcomes, and survival curve to those in singleton pregnancies. Furthermore, we found that preoperative CL ≤ 15 mm was independently associated with preterm birth before 34 weeks of gestation according to multivariate regression analysis, rather than twin gestation itself and prior preterm history, which were common risk factors listed in other studies [1, 7, 22]. These findings highlight that a CL ≤ 15 mm is important risk factor for predicting preterm birth in twin pregnancies, consistent with the findings of previous studies [7, 17, 18].

This study's strengths include having been the largest retrospective cohort study to evaluate the outcomes of UIC in twin pregnancies in a single center. Further, because all procedures were performed by two maternal-fetal medicine specialists at our center, there were fewer interoperator variations and confounding variations in all procedures. In addition, our study suggested a definite cut-off value (CL ≤ 15 mm) of cervical length through ROC curve analysis and proved that a certain CL (≤15 mm) was an important risk factor for predicting preterm delivery through various analyses.

However, this study also has several limitations. First, this was a retrospective study, therefore, selection bias was possible. Second, the sample size was relatively small, so the possibility of a type II error could not be ruled out. Third, we designated singleton pregnancies that underwent UIC as a control group instead of twin pregnancies that did not receive UIC. Although singleton pregnancies with a previous preterm birth history or second trimester loss are appropriate candidates for cerclage, this comparison between singleton and twin pregnancies is an indirect method to evaluate the effectiveness of cerclage in twin pregnancies.

To overcome these limitations, large, randomized trials are needed to represent the overall population to assess the outcomes of twin UIC. Further research is recommended to investigate the influence of confounding variables on pregnancy and neonatal outcomes.

## 5. Conclusion

Our study provides promising results regarding the effectiveness and safety of UIC in twin pregnancies with short cervices. The rates of preterm birth before 32, 28, and 24 weeks in twin pregnancies, neonatal mortalities, and morbidities were comparable to those in singleton pregnancies. Furthermore, a preoperative CL ≤ 15 mm may be an important prognostic factor associated with preterm birth before 34 weeks, and UIC in twin pregnancies with a CL ≤ 15 mm was associated with pregnancy and neonatal outcomes comparable to those of singleton pregnancies. These results are clinically important as they may provide clinicians with accurate data on the prognosis of UIC in twin pregnancies and guide patients counselling.

## Figures and Tables

**Table 1:** Comparison of baseline characteristics based on singleton or twin pregnancy.

Parameter study population (*N* = 94)	Singleton pregnancy (*N* = 56)	Twin pregnancy (*N* = 38)	*P* value^∗^
Maternal age (years)	34 (19–43)	33 (25–38)	0.01^∗^
Parity			<0.001^∗^
Primiparous	0 (0)	28/38 (73.7)	
Multiparous	56 (100)	10/38 (26.3)	
ART	4 (7.1)	26 (68.4)	<0.001^∗^
Prior preterm birth or second trimester loss	56 (100)	2 (5.3)	<0.001^∗^
Prior cervical surgery	4 (7.1)	1 (2.6)	0.65
BMI	24.7 (18.1–33.5)	24.1 (18.0–40.4)	0.93
GA at cerclage (weeks)	21.4 (16.4–23.6)	21.4 (16.3–23.4)	0.64
Use of tocolytics	22 (39.3)	21 (55.3)	0.14
Use of vaginal progesterone	56 (100)	36 (94.7)	0.08

Data are expressed as medians (ranges) and number (percentage). ^∗^*P* < 0.05, which means statistical difference. ART: assisted reproductive technology; BMI: body mass index; GA: gestational age.

**Table 2:** Comparison of pregnancy outcomes based on singleton or twin pregnancy.

Pregnancy outcomes study population (*N* = 94)	Singleton pregnancy (*N* = 56)	Twin pregnancy (*N* = 38)	*P* value^∗^
Preoperative cervical length (mm)	13.6 (4.0–24.0)	14.6 (3.5–25.0)	0.65
GA at delivery (weeks)	36.1 (25.5–40.4)	32.6 (23.6–37.4)	0.004^∗^
Cerclage to delivery interval (days)	101 (23–152)	87 (20–141)	0.01^∗^
Preterm delivery			
<34 weeks	15 (26.8)	20 (52.6)	0.02^∗^
<32 weeks	15 (26.8)	16 (42.1)	0.18
<28 weeks	6 (10.7)	6 (15.8)	0.54
<24 weeks	2 (3.6)	1 (2.6)	1

Data are expressed as medians (ranges) and number (percentage). ^∗^*P* < 0.05, which means statistical difference. GA: gestational age.

**Table 3:** Comparison of neonatal outcomes based on singleton or twin pregnancy.

Neonatal outcomes study population (*N* = 132)	Singleton pregnancy (*N* = 56)	Twin pregnancy (*N* = 38*×*2 = 76)	*P* value^∗^
Birth weight (g)	2750 (930 ─ 3760)	1920 (530 ─ 3630)	<0.001^∗^
Very low birth weight	14 (25.0)	19 (25.0)	1
Extremely low birth weight	3 (5.4)	8 (10.5)	0.35
Apgar score at 1 minute (<7)	18 (32.1)	43 (56.6)	0.008^∗^
Apgar score at 5 minutes (<7)	6 (10.7)	18 (23.7)	0.07
Mortality	1 (1.8)	3 (3.9)	0.64
Immediate death	1 (1.8)	0 (0)	0.42
<7days	0 (0)	2 (2.6)	0.51
>7days	0 (0)	1 (1.3)	1
Respiratory distress syndrome	12 (21.4)	21 (27.6)	0.54
Bronchopulmonary dysplasia	7 (12.5)	11 (14.5)	0.81
Necrotizing enterocolitis	0 (0)	1 (1.3)	1
Intraventricular hemorrhage	1 (1.8)	8 (10.5)	0.08
Retinopathy of prematurity	10 (17.9)	23 (30.3)	0.154
Sepsis	9 (16.1)	23 (30.3)	0.07

Data are expressed as medians (ranges) and number (percentage). ^∗^*P* < 0.05, which means statistical difference.

**Table 4:** Multivariable analysis of factors associated with preterm birth before 34 weeks.

Variables	Odds ratio	95% CI	*P* value^∗^
Twin gestations	0.336	0.018–6.331	0.47
History of prior preterm birth or second trimester loss	1.059	0.056–20.197	0.97
Preoperative cervical length ≤ 15 mm	2.870	1.150–7.164	0.02^∗^

CI: confidence interval. ^∗^*P* < 0.05, which means statistical difference.

**Table 5:** Comparison of pregnancy outcomes based on singleton or twin pregnancy with preoperative cervical length ≤ 15 mm.

Pregnancy outcome study population (*N* = 50)	Singleton pregnancy (*N* = 29)	Twin pregnancy (*N* = 21)	*P* value^∗^
GA at cerclage (weeks)	21.5 (16.4–23.6)	21.3 (17.2–23.4)	0.91
GA at delivery (weeks)	35.1 (25.5–40.4)	32.5 (23.6–37.4)	0.24
Preoperative cervical length (mm)	9.0 (4.0–13.6)	10.0 (3.5–15.0)	0.46
Postoperative cervical length (mm)	27.4 (11.7–40.4)	25.0 (17.7–41.8)	0.81
Cerclage to delivery interval (days)	83 (23–152)	82 (20–115)	0.43
Preterm delivery			
<34 weeks	12 (41.4)	12 (57.1)	0.39
<32 weeks	12 (41.4)	9 (42.9)	1
<28 weeks	6 (20.7)	3 (14.3)	0.72
<24 weeks	2 (6.9)	1 (4.8)	1

Data are expressed as medians (ranges) and number (percentage). ^∗^*P* < 0.05, which means statistical difference. GA: gestational age.

**Table 6:** Comparison of neonatal outcomes based on singleton or twin pregnancy with preoperative cervical length ≤ 15 mm.

Neonatal outcome study population (*N* = 71)	Singleton pregnancy (*N* = 29)	Twin pregnancy (*N* = 21*×*2 = 42)	*P* value^∗^
Birth weight (g)	2870 (930–3630)	1835 (530–3630)	0.04^∗^
Very low birth weight	12 (41.4)	12 (28.6)	0.31
Extremely low birth weight	3 (10.3)	6 (14.3)	0.73
Apgar score at 1 minute (<7)	13 (44.8)	28 (66.7)	0.09
Apgar score at 5 minutes (<7)	5 (17.2)	10 (23.8)	0.57
Mortality	1 (3.4)	2 (4.8)	1
Immediate death	1 (3.4)	0 (0)	0.41
<7days	0 (0)	1 (2.4)	1
>7days	0 (0)	1 (2.4)	1
Respiratory distress syndrome	10 (34.5)	11 (26.2)	0.60
Bronchopulmonary dysplasia	7 (24.1)	5 (11.9)	0.21
Necrotizing enterocolitis	0 (0)	1 (2.4)	1
Intraventricular hemorrhage	1 (3.4)	6 (14.3)	0.23
Retinopathy of prematurity	8 (27.6)	17 (40.5)	0.32
Sepsis	7 (24.1)	13 (31.0)	0.60

Data are expressed as medians (ranges) and number (percentage). ^∗^*P* < 0.05, which means statistical difference.

## Data Availability

The data and materials in this study are available from the corresponding author on request.
